# Differential genome-wide profiling of alternative polyadenylation sites in nasopharyngeal carcinoma by high-throughput sequencing

**DOI:** 10.1186/s12929-018-0477-6

**Published:** 2018-10-23

**Authors:** Ya-Fei Xu, Ying-Qing Li, Na Liu, Qing-Mei He, Xin-Ran Tang, Xin Wen, Xiao-Jing Yang, Ying Sun, Jun Ma, Ling-Long Tang

**Affiliations:** 1 Sun Yat-sen University Cancer Center; State Key Laboratory of Oncology in South China; Collaborative Innovation Center for Cancer Medicine; Guangdong Key Laboratory of Nasopharyngeal Carcinoma Diagnosis and Therapy, Guangzhou, 510060 People’s Republic of China; 2 Department of Cell Biology and Genetics, Shenzhen University Health Science Center, Shenzhen, 518060 People’s Republic of China

**Keywords:** Alternative polyadenylation, Genome-wide profiling, High-throughput sequencing, Nasopharyngeal carcinoma

## Abstract

**Background:**

Alternative polyadenylation (APA) is a widespread phenomenon in the posttranscriptional regulation of gene expression that generates mRNAs with alternative 3′-untranslated regions (3’UTRs). APA contributes to the pathogenesis of various diseases, including cancer. However, the potential role of APA in the development of nasopharyngeal carcinoma (NPC) remains largely unknown.

**Methods:**

A strategy of sequencing APA sites (SAPAS) based on second-generation sequencing technology was carried out to explore the global patterns of APA sites and identify genes with tandem 3’UTRs in samples from 6 NPC and 6 normal nasopharyngeal epithelial tissue (NNET). Sequencing results were then validated using quantitative RT-PCR in a larger cohort of 16 NPC and 16 NNET samples.

**Results:**

The sequencing data showed that the use of tandem APA sites was prevalent in NPC, and numerous genes with APA-switching events were discovered. In total, we identified 195 genes with significant differences in the tandem 3’UTR length between NPC and NNET; including 119 genes switching to distal poly (A) sites and 76 genes switching to proximal poly (A) sites. Several gene ontology (GO) terms were enriched in the list of genes with switched APA sites, including regulation of cell migration, macromolecule catabolic process, protein catabolic process, proteolysis, small conjugating protein ligase activity, and ubiquitin-protein ligase activity.

**Conclusions:**

APA site-switching events are prevalent in NPC. APA-mediated regulation of gene expression may play an important role in the development of NPC, and more detailed studies targeting genes with APA-switching events may contribute to the development of novel future therapeutic strategies for NPC.

**Electronic supplementary material:**

The online version of this article (10.1186/s12929-018-0477-6) contains supplementary material, which is available to authorized users.

## Background

Nasopharyngeal carcinoma (NPC) is an epithelial malignancy of the head and neck with a highly unbalanced ethnic and geographic distribution. It occurs mainly in Southern China and Southeast Asia, where the incidence can be as high as 20 to 50 cases per 100,000 person-years [1–3]. Although the 5-year overall survival rate is approximately 60% [4] and has increased with the advent of intensity-modulated radiation therapy [5], the prognosis of NPC is still very poor due to recurrence or/and distant metastasis [6]. The etiology of NPC is multi-factorial; including genetic components, Epstein-Barr virus (EBV) infection, environmental factors, and interactions between these factors [7–9]. Among these factors, genetic components play a vital role in the development of NPC and various genes related to NPC have been identified [10–13]. However, genomic abnormalities in NPC tumorigenesis remain largely unidentified. It is particularly important that the genomic foundations of NPC are clearly defined to identify genes contributing to the initiation and progression of NPC, and further guide the development of novel therapeutic strategies for NPC patients.

Posttranscriptional regulation at the mRNA level is generally mediated by miRNAs and RNA-binding proteins which recognize and bind to elements within the 3′-untranslated region (3’UTR). Dysregulation of this can perturb gene expression and contribute to the pathogenesis of various diseases, including several types of cancer [14, 15]. The eukaryotic transcriptome is particularly complicated due to its use of alternative polyadenylation (APA) sites, which can generate diverse mRNA isoforms from a single gene that differ either in their coding sequence, their 3’UTRs, or both [16]. APA is a widespread phenomenon, with more than half of the genes in humans and over 30% in mice having numerous APA sites [17]. Recent studies have shown that APA-switching events may occur in a tissue- or disease-specific manner [18]. The mRNA transcripts in placenta, ovaries and blood are characterized by preferential usage of proximal poly(A) sites (generating isoforms with shorter 3’UTRs), whereas in nervous system and brain, they tend to use distal poly(A) sites (generating isoforms with longer 3’UTRs) [19].

Tandem 3’UTRs can also affect mRNA stability, translation efficiency, and subcellular localization by causing loss of regulatory elements, especially miRNA binding sites in the 3’UTR [20–22]. APA-switching events can influence a number of critical biological processes, including embryonic development and cell differentiation [23–25], cell proliferation [26, 27], immune response [22], neuron activation [28, 29], and tumorigenesis [27, 30–32]. It has been shown that activated T lymphocytes [22] and cancer cells [27] generally use shorter 3’UTRs, and that shortening of tandem 3’UTRs is associated with cell proliferation [22] while the lengthening of tandem 3’UTRs has been observed during cell differentiation [24, 33]. study by Mayr et al. showed that shortening of tandem 3’UTRs was preferentially used by several oncogenes in cancer cell lines [27], suggesting APA-mediated gene expression may play an important role in cancer development. Recent studies also reported that APA-switching events occurred in various types of cancer, including breast cancer [30], colorectal carcinoma [32], glioblastoma [34], and gastric cancer [35]. Nevertheless, whether APA-switching events are integral to the development and progression of NPC remains unknown.

In this study, for the first time, we performed genome-wide profiling of APA sites, with a sequencing APA sites (SAPAS) technology using second-generation sequencing, in NPC and normal nasopharyngeal epithelial tissues (NNET) to identify genes with 3’UTR switching that participate in NPC development. Gene Ontology (GO) and pathway analysis were performed to better understand the function of genes with 3’UTR switching. Subsequently, we validated our sequencing results by quantitative RT-PCR in a larger sample size. Our research provides a novel insight into the tumorigenesis of NPC.

## Methods

### Clinical specimens

Sixteen NPC fresh tissue samples were collected at the Department of Radiation Oncology of Sun Yat-sen University Cancer Center (Guangzhou, China) between 16 Jan 2010 and 25 Feb 2013. All samples were reassessed by two pathologists and the percentage of tumor cells was 70% or more in all samples. None of the patients had received radiotherapy or chemotherapy before biopsy sampling. 16 NNET tissue samples were collected from outpatients, showed no evidence of cancer, and the tissue samples showed normal histology. This study was approved by the Human Ethics Approval Committee at Sun Yat-sen University Cancer Center. Written informed consent was obtained from each study subject.

### RNA extraction

Total RNA was extracted using TRIzol reagent (Life Technologies, Grand Island, NY, USA) according to the manufacturer’s instructions. Genomic DNA was removed from total RNA by TURBO DNase (Ambion, Austin, TX, USA). The quantity of RNA was determined using a NanoDrop2000 spectrophotometer (Thermo Scientific, Wilmington, DE, USA) and RNA purity was assessed by the ratio of absorbance at 260 and 280 nm. RNA quality was assessed using electrophoresis in a 1.5% agarose gel stained with ethidium bromide.

### Construction of the 3’UTR library

The SAPAS sequencing libraries were constructed as described previously [30, 36]. In brief, total RNA was randomly fragmented by heating. The first strand cDNA was generated by a template-switch reverse transcription (RT) reaction, in which contains an anchored oligo d(T) primer, a 5′ template switching adaptor, and Super-Script II kits (Invitrogen Life Technologies, Karlsruhe, Germany). Then, ds-cDNA was amplified by PCR using known primers tagged with Illumina adaptors. The sequences of all primers were consistent with previous studies [30, 37]. PAGE gel-excision was carried out to select the PCR products with the fragments of 300-500 bp with a QIAquick Gel Extraction Kit (Qiagen, Valencia, CA). The final pooled fragments were sequenced from their 3’end on the Illumina HiSeq 2000 system in dark cycle mode. Finally 58 bp reads were generated.

### Illumina reads filtering and mapping

Reads were filtered and trimmed with in-house Perlscripts, and the trimmed reads were aligned to the human genome (hg19) with Bowtie. Internal priming filtering was performed by analyzing the genomic sequences located 1 to 20 bases downstream of poly(A) cleavage sites, containing the sequence motifs 5′-AAAAAAAA-3′, 5′-GAAAA+GAAA+G-3′ (“+” means “or more”), or more than 12 “A”s. Sites with only one read were removed.

### Poly(A) sites identification and annotation

Cleavage sites were clustered into poly(A) sites as described previously [17, 30]. Briefly, the reads of which 3′ ends located within 24 nt from each other and which aligned to the same strand of a chromosome were clustered. Then, cleavage clusters with two or more reads were assigned as poly(A) sites. Next, poly(A) sites were grouped into three types according to the known poly(A) sites in the UCSC transcript ends database [38] and Tian’s database [17]: 1) UCSC known genes, where the poly(A) site was located within 24 nt from the 3′ transcription end of a UCSC gene; 2) Tian poly(A) DB, where the poly(A) site was located within 24 nt from a poly(A) site in Tian’s poly(A) DB; 3) Putative novel poly(A) sites, containing the following six attributes: 3’UTR; located <= 1 kb nt downstream of a UCSC gene; CDS; intron; intergenic; and noncoding gene. Meanwhile, poly(A) sites with reads that overlapped Ensemble-annotated 3’UTR(s) were defined as tandem poly(A) sites. The expression of mRNAs were accumulated by reads of all poly(A) sites, and then the differential expressed genes were identified by edgeR [39]. The raw sequencing data can be accessed from the NCBI Bioproject (http://www.ncbi.nlm.nih.gov/bioproject) under accession no. PRJNA299088.

### 3’UTR switching analysis between NPC and NNET and functional annotation

3’UTR switching of each gene between 6 NPC and 6 NNET tissues was detected by a pair-wise case-control analysis, which sequentially compared NPC samples to NNET samples to identify genes with 3’UTR switching in each pair. They were then filtered using the Benjamini-Hochberg false discovery rate (FDR), whereby those estimated to be below 0.01 (using R software, version 2.15) were kept. In total, genes with 3’UTR switching that occurrences in more than 10 pairs were defined as genes with tandem 3’UTR between NPC and NNET. Functional annotation analysis of these genes was performed using DAVID Bio-informatics Resources (http://david.abcc.ncifcrf.gov/) [40]. The miRNA targets involved in APA-switching events were identified by TargetScan database [41].

### Validation of quantitative RT-PCR analysis

To validate the sequencing data, quantitative RT-PCR was carried out in 16 NPC and 16 NNET tissues for eight genes (*JAG1*, *IRF1*, *EGLN1*, *TIMP3*, *WDR5*, *SMAD3*, *FNDC3B*, and *XRCC5*) with extreme 3’UTR length differences between NPC and NNET. Based on SAPAS data, the poly(A) sites of each gene were divided into two supersites: “proximal sites” and “distal sites”. Two gene-specific primer sets were specifically designed for “proximal sites” and “distal sites”, as described previously [30]. Primer sequences are listed in Additional file 1. The quantitative RT-PCR was performed on the CFX96TouchTM sequence detection system (Bio-Rad, Hercules, CA, USA) using Platinum SYBR Green qPCR SuperMix-UDG reagents (Invitrogen) according to the manufacturer’s instructions. GAPDH was used as a control for normalization. For each gene, the relative expression ratio of the proximal site to the distal site was calculated using the 2^-ΔΔCT^ method [42].

### Statistical analysis

SPSS 16.0 software was used for statistical analysis. All of the data were presented as the mean ± SD. The Student’s t-test was used to determine whether significant differences existed in the usage of poly(A) sites of genes between two groups, and two-tailed *P* < 0.05 was considered significant.

## Results

### Global deep sequencing of 3′ ends of mRNA

We used the SAPAS strategy to profile APA sites of 6 NPC and 6 NNET tissues. In total, 20.99 to 26.97 million raw reads with lengths of 58 bp were obtained from Illumina sequencing. A statistical summary of the data is shown in Table 1. Approximately 20.83 to 26.64 million reads harbored the modified anchor oligo d(T), of which 13.02 to 15.71 million reads uniquely mapped to the human nucleus genome (hg19) [43]. Furthermore, 9.83 to 12.68 million reads that could be used directly to infer transcript cleavage sites were obtained after filtering the reads with internal priming. In total, 21,095 UCSC canonical genes were sequenced by at least one read, which accounted for 27.2% of all canonical genes.

**Table 1:** Summary of the SAPAS data from Illumina HiSeq 2000 sequencing

	NPC						NNET					
	NPC1	NPC2	NPC3	NPC4	NPC5	NPC6	NNET1	NNET2	NNET3	NNET4	NNET5	NNET6
Raw reads(M*)	26.08	23.81	24.48	25.29	25.14	20.99	25.94	23.91	25.77	24.87	26.97	24.56
Clean reads(M)	25.90	23.54	24.06	24.94	24.94	20.83	25.77	23.59	25.33	24.70	26.64	24.42
Mapped to genome(M)	21.73	19.75	20.02	21.06	21.39	17.45	21.28	19.49	21.09	19.53	19.46	21.10
Uniquely mapped to genome(M)	17.02	16.42	16.11	16.82	17.29	14.33	17.45	15.87	17.75	16.17	15.72	17.48
Mapped to nuclear genome(M)	13.32	14.63	13.11	13.02	14.69	13.34	15.21	14.45	15.71	14.57	13.92	14.76
Passed Internal Priming filter(M)	11.51	9.83	10.18	10.30	12.68	11.36	12.00	11.47	10.01	12.50	12.06	12.36
Genes sampled by reads(10 K)	1.78	1.89	1.88	1.90	1.80	1.83	1.86	1.88	1.91	1.88	1.73	1.87
Poly(A) sites(10 K)	13.70	29.99	17.36	19.04	13.82	12.50	22.75	18.14	36.03	16.28	12.45	14.90
Known poly(A) sites sampled(10 K)	2.75	2.90	2.89	2.89	2.82	2.87	2.84	2.92	2.86	2.97	2.67	2.90
Putative novel poly(A) sites(10 K)	10.95	27.09	14.47	16.15	11.00	9.63	19.91	15.21	33.17	13.31	9.78	12.01
Genes sampled by poly(A) sites(10 K)	1.62	1.72	1.71	1.73	1.63	1.66	1.73	1.72	1.73	1.72	1.53	1.72

### A comprehensive inventory of poly(A) sites

**Fig. 1:**
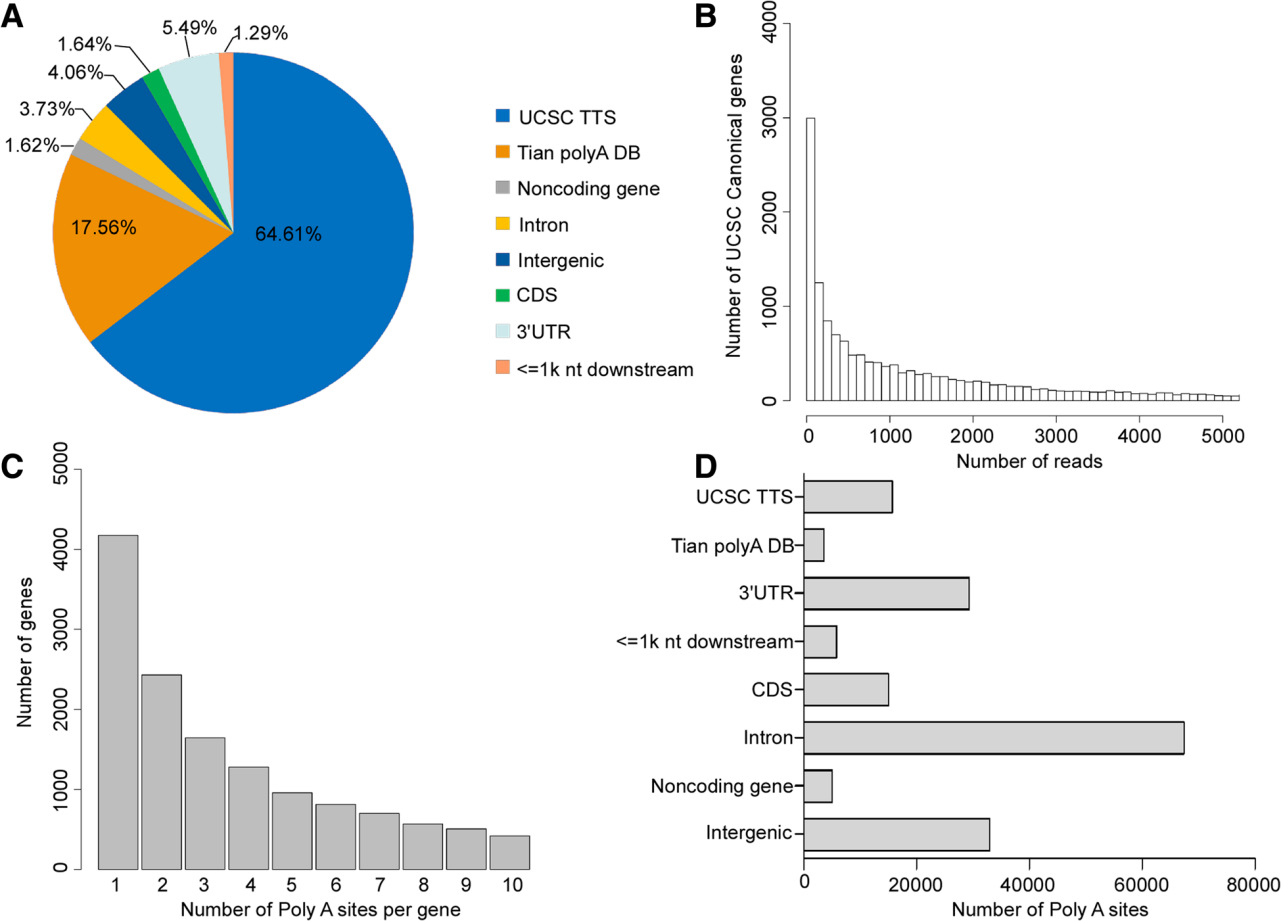
The characteristics of the SAPAS data.** a** The genomic locations of reads that specifically aligned to the nuclear genome after internal priming filtering. **b** The histogram of the number of reads for UCSC canonical genes. **c** The distribution of numbers of poly(A) sites per gene. **d** The genomic locations of poly(A) sites for UCSC canonical genes

As shown in Fig. 1a, the majority of filtered reads (82.17%) were mapped to known poly(A) sites listed in the UCSC transcripts ends database [38] and Tian’s database [17]. An additional 5.49% and 1.29% of reads were mapped to the 3’UTR and 1 kb region downstream from the UCSC canonical genes, respectively. The distribution of the number of all reads is shown in Fig. 1b. As a result of the heterogeneity of cleavage sites at poly(A) sites, we took the cleavage clusters with more than one read as poly(A) sites. We observed that 13,181 genes had more than one tandem APA site, among which 10,753 genes harbored more than two tandem APA sites (Fig. 1c). In total, 174,931 poly(A) sites were identified from all twelve samples. Only 11.04% of these sites were found in the UCSC and Tian databases. Another 16.78% of the poly(A) sites were found in the 3’UTRs, 3.31% within 1 kb downstream from the UCSC canonical genes,18.86% in intergenic, 38.57% in intron, 2.87% in noncoding genes, and 8.56% in CDS from the UCSC canonical genes (Fig. 1d).

### Differential usage of poly(A) between NPC and NNET

Previous studies have demonstrated that most cancer cells [27] and cancer tissues [31, 32] tend to use shortened 3’UTRs. In contrast to these results for primary cancers, however, one study reported that a greater number of genes with lengthened 3’UTRs existed in a metastasis cell line [30]. In our study, the 3’UTR length of each gene was calculated according to the distance between the poly(A) sites and the stop codon. Subsequently, a pair-wise case-control analysis between NPC and NNET tissues was performed, and a total of 36 pairs were obtained. 3’UTR switching for each gene within each pair was detected using a test of linear trend alternative to independence [44]. A positive Pearson correlation coefficient (r) indicates that NPC tissue contains more lengthened tandem 3’UTRs than NNET, while a negative indicates that NPC tissue harbors more shortened tandem 3’UTRs. The genes with 3’UTR switching between NPC and NNET obtained for each pair are shown in Table 2. We further analyzed the 3’UTR switching frequency for each gene in 36 pairs and found that the frequency of the genes with 3’UTR switching was the highest in at least 10 pairs (Additional file 2). Therefore, we set 10 pairs as the selection criteria for further screening of genes with 3’UTR switching. As a result, we identified 195 genes with significantly different 3’UTR lengths (false discovery rate [FDR] < 0.01, *P* < 0.01), of which 119 genes switched to longer 3’UTRs and 76 genes switched to shorter 3’UTRs in NPC. Details of the 195 genes with significant differences between NPC and NNET are shown in Additional file 3.

**Table 2:** Genes with APA site switching between NPC and NNET tissues using a pair-wise case-control analysis

Pairs	3’UTR shortened genes	3’UTR lengthened genes	Combined
NPC1 vs. NNET1	104	372	476
NPC1 vs. NNET2	179	192	371
NPC1 vs. NNET3	144	247	391
NPC1 vs. NNET4	58	242	300
NPC1 vs. NNET5	99	446	545
NPC1 vs. NNET6	51	123	174
NPC2 vs. NNET1	55	85	140
NPC2 vs. NNET2	91	13	104
NPC2 vs. NNET3	108	38	146
NPC2 vs. NNET4	100	85	185
NPC2 vs. NNET5	123	234	357
NPC2 vs. NNET6	184	106	290
NPC3 vs. NNET1	63	143	206
NPC3 vs. NNET2	104	23	127
NPC3 vs. NNET3	51	26	77
NPC3 vs. NNET4	79	103	182
NPC3 vs. NNET5	68	178	246
NPC3 vs. NNET6	149	111	260
NPC4 vs. NNET1	62	167	229
NPC4 vs. NNET2	210	104	314
NPC4 vs. NNET3	136	115	251
NPC4 vs. NNET4	158	236	394
NPC4 vs. NNET5	156	355	511
NPC4 vs. NNET6	133	125	258
NPC5 vs. NNET1	158	377	535
NPC5 vs. NNET2	193	116	309
NPC5 vs. NNET3	150	153	303
NPC5 vs. NNET4	55	112	167
NPC5 vs. NNET5	107	286	393
NPC5 vs. NNET6	66	82	148
NPC6 vs. NNET1	102	202	304
NPC6 vs. NNET2	137	24	161
NPC6 vs. NNET3	147	99	246
NPC6 vs. NNET4	53	58	111
NPC6 vs. NNET5	90	172	262
NPC6 vs. NNET6	139	76	215

### Functional annotation analysis of the genes with switched APA sites

To explore the biological significance of these APA site-switching genes, a functional annotation of the above 195 genes was performed with Database for Annotation, Visualization and Integrated Discovery (DAVID) Bio-informatics Resources, where single-UTR genes were set as the background. The results of Gene Ontology (GO) terms analysis showed that genes with tandem 3’UTRs were mainly enriched in the regulation of cell migration, macromolecule catabolic process, protein catabolic process, proteolysis, small conjugating protein ligase activity, and ubiquitin-protein ligase activity (Table 3). Additionally, genes with tandem 3’UTRs involved in the ubiquitin-mediated proteolysis pathway (*P* = 0.022), lysosomepathway (*P* = 0.048), colorectal cancer(*P* = 0.017) and renal cell carcinoma pathway (*P* = 0.049) were enriched (Table 3). We also performed GO analysis and pathway enrichment analysis by separating the 195 genes into two groups (switching to distal poly(A) and proximal poly(A)), and results were showed in Additional files 4, 5. Since distant metastasis occurs in 20–30% of NPC patients and is the major cause of death in NPC [6], it is noteworthy that we identified 9 genes with enriched tandem 3’UTRs that are involved in the regulation of cell migration; namely *SMAD3*, *JAG1*, *Pikr1*, *Ptp4a1*, *Rac1*, *RRAS2*, *Spag9*, *TRIP6*, and *TRIB1* (Table 4). These results indicate APA site-switching events can influence a number of critical biological processes and may play an important role in the development of NPC.

**Table 3:** Enrichment of genes with tandemed 3’UTR isoforms involved in various GO functional categories

GO category		Count	*P*-value
GOTERM_BP_FAT	Regulation of cell migration	9	0.0004
GOTERM_BP_FAT	Regulation of locomotion	9	0.001
GOTERM_BP_FAT	Regulation of cell motion	9	0.001
GOTERM_BP_FAT	Macromolecule catabolic process	19	0.0015
GOTERM_BP_FAT	Protein catabolic process	16	0.0024
GOTERM_BP_FAT	Proteolysis	20	0.015
GOTERM_MF_FAT	Small conjugating protein ligase activity	7	0.0052
GOTERM_MF_FAT	Acid-amino acid ligase activity	7	0.013
GOTERM_MF_FAT	Ubiquitin-protein ligase activity	6	0.013
KEGG_PATHWAY	Ubiquitin mediated proteolysis	6	0.022
KEGG_PATHWAY	Lysosome	5	0.048
KEGG_PATHWAY	Colorectal cancer	5	0.017
KEGG_PATHWAY	Renal cell carcinoma	4	0.049

**Table 4:** Nine genes enriched in GO terms associated with cell migration

UCSC ID	Gene Symbol	Gene Name	*r*-value
uc002aqj.2	SMAD3^a^	SMAD family member 3	0.086
uc002wnw.2	JAG1^a^	jagged 1 (Alagille syndrome)	−0.351
uc003jva.2	Pikr1	phosphoinositide-3-kinase, regulatory subunit 1 (alpha)	0.0007
uc003pek.2	Ptp4a1	protein tyrosine phosphatase type IVA, member 1	0.929
uc003spw.2	Rac1	ras-related C3 botulinum toxin substrate 1 (rho family, small GTP binding protein Rac1)	0.175
uc009ygq.2	RRAS2	related RAS viral (r-ras) oncogene homolog 2; similar to related RAS viral (r-ras) oncogene homolog 2	− 0.564
uc002ita.2	Spag9	sperm associated antigen 9	0.411
uc003uww.2	TRIP6	thyroid hormone receptor interactor 6	0.182
uc003yrx.2	TRIB1	tribbles homolog 1 (Drosophila)	−0.206

^a^genes selected for quantitative RT-PCR validation

*r*-value: a positive value of r indicates a longer tandem 3’UTR in NPC, and vice versa

### The effects of APA-site switching events on gene expression and miRNA targeting sites

To further explore the functional effects of APA-site switching events, we firstly analyzed the relationship between the APA switched sites and the mRNA levels of 195 genes. A total of 275 genes with significant differences in the mRNA levels between NPC and NNET were identified, including 198 genes that were up-regulated and 77 genes that were down-regulated in NPC (Additional file 6). Further analyses revealed that among the 119 genes that switched to longer 3’UTRs in NPC, 4 genes were up-regulated and none was down-regulated; and among the 76 genes that switched to shorter 3’UTRs, only one gene was found to be up-regulated or down-regulated in NPC (Additional file 7). These results suggest that the APA-site switching events could not change the mRNA expression levels, and it may affect the translation efficiency and subcellular localization by causing loss of regulatory elements, especially miRNA binding sites in the 3’UTR.

Furthermore, we identified potential miRNA targeting sites between the “proximal polyA sites” and “distal polyA sites” of 195 genes using the publicly available databases TargetScan to explore the effect of APA-switching events on the gain-of or loss-of miRNA targeting sites in their 3’UTR. The results showed that for the 119 genes switched to longer 3’UTRs in NPC, totally 706 miRNA targeting sites were gained and for the 76 genes switched to shorter 3’UTRs in NPC, totally 325 miRNA targeting sites were lost (Additional file 8). These result suggests that APA-switching events can result in the gain or loss of miRNA binding sites in the 3’UTR in the development of NPC.

### Real-time RT-PCR validation of selected APA switched sites

**Fig. 2:**
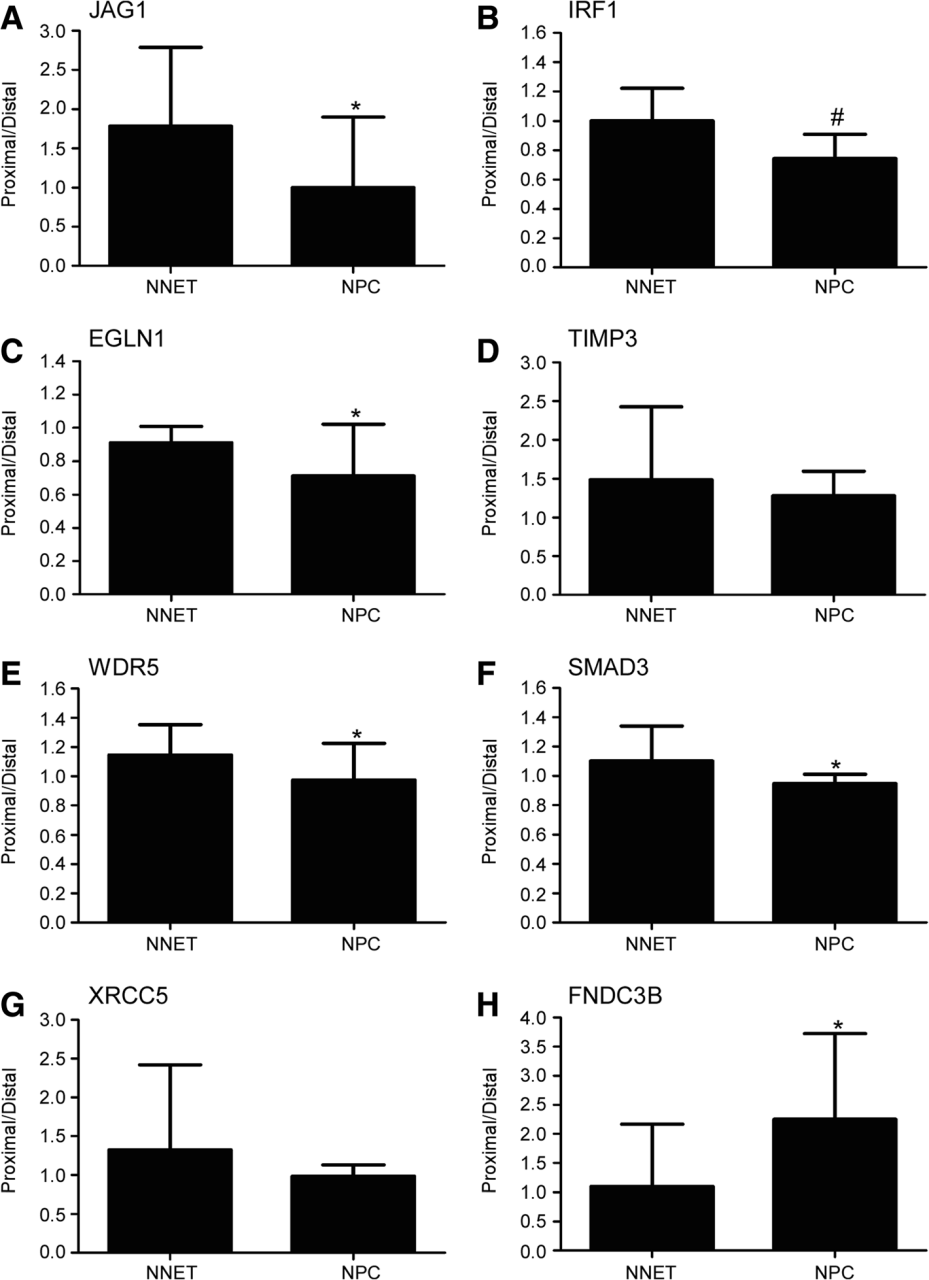
Validation of 3’UTR switching in 16 NPC and NNET samples with quantitative RT-PCR. **a** *JAG1*; (B) *IRF1*; (**c**) *EGLN1*; (**d**) *TIMP3*; (**e**) *WDR5*; (**f**) *SMAD3*; (**g**) *XRCC5*; (**h**) *FNDC3B*. Proximal/Distal indicates the expression ratio of the shortened 3’UTR to the lengthened 3’UTR

To further validate the SAPAS sequencing data, we randomly selected eight genes (*JAG1*, *IRF1*, *EGLN1*, *TIMP3*, *WDR5*, *SMAD3*, *FNDC3B*, and *XRCC5*) with switched APA sites for quantitative RT-PCR validation in 16 NPC and 16 NNET samples. Results of six genes were similar to the sequencing data (Fig. 2). *JAG1*, *IRF1*, *EGLN1*, *WDR5*, and *SMAD3* tended to use lengthened 3’UTR transcripts, whereas *FNDC3B* tended to use shortened 3’UTR transcripts (*P* < 0.05). Consistent with the sequencing data, there appeared to be a higher tendency for lengthened 3’UTR transcripts from the genes *TIMP3* and *XRCC5* to be present in NPC than NNET, however no statistical differences were observed. These tendencies suggest that APA-switching events were more prevalent in NPC tissues.

## Discussion

APA allows a single gene to encode multiple mRNA transcripts by changing the mRNA 3’UTR length and plays an important role in various physiological and pathological processes, including tumorigenesis [27, 30, 45, 46]. In this study, we performed genome-wide profiling of tandem APA sites in NPC and NNET tissues using SAPAS based on second-generation sequencing technology. In total, we identified 195 genes whose tandem 3’UTR length differed significantly between NPC and NNET, including 119 genes switching to distal poly(A) sites and 76 genes switching to proximal poly(A) sites. Several gene ontology (GO) terms were enriched in the list of genes with switched APA sites, including regulation of cell migration, macromolecule catabolic process, protein catabolic process, proteolysis, small conjugating protein ligase activity, and ubiquitin-protein ligase activity. The sequencing results were further validated using quantitative RT-PCR.

APA, which leads to alterations in 3’UTR length and content of genes, shows dynamic characteristics in a variety of tumors. A previous analysis of 27 cancer cell lines derived from sarcomas and breast, lung and colon cancers showed that tumor cells generally use the shorter 3’UTRs [27]. Similarly, Lin et al. found that shorter 3’UTRs isoforms were preferentially upregulated in breast, colon, kidney, liver and lung cancer tissues [31]. Likewise, Morriset al. demonstrated that more genes tended to use shorter 3’UTRs isoforms in colorectal carcinoma compared to colorectal adenoma and normal colon mucosa [32]. However, a recent discovery showed that longer 3’UTRs isoforms were significantly upregulated in MB231, a human breast cancer line that is estrogen independent and highly invasive [30]. Nordlund et al. demonstrated that more genes preferentially used longer 3’UTRs in acute lymphoblastic leukemia [47]. These findings suggest that APA-switching events are more complicated and may occur in a tissue- or disease-specific manner. In this study, we found that 119 genes tended to use longer 3’UTRs and 76 genes tended to use shorter 3’UTRs in NPC, which is the first genome-wide research to investigate APA in NPC.

Furthermore, 3’UTRs harbor several cis-elements, such as U-rich elements (USE), poly A signals (PAS), ARE (AU-rich elements), cytoplasmic polyadenylation elements, miRNA target sites and other unknown elements [46]. Therefore, APA-induced alterations in 3’UTR length and content may result in loss or gain of these regulatory motifs, resulting in series of changes in cell biological function by affecting mRNA stability, transcript export and translation efficiency [48]. Recent studies have revealed that shorter 3’UTRs could lead to greater mRNA stability and increased protein output, and were also associated with elevated cell proliferation rates or transformation [27]. In this study, we identified 195 genes with tandem 3’UTRs isoforms, which were involved in various cellular biological functions, including regulation of cell migration, macromolecule catabolic processes, protein catabolic processes, proteolysis, small conjugating protein ligase activity, and ubiquitin-protein ligase activity. This result is not unexpected because it has been confirmed that metastasis occurs in 20–30% of NPC patients and is the major cause of death in NPC [6]. It is important to note that our study effectively identified genes that were involved in NPC tumorigenesis.

To date, several possible mechanisms have been reported to influence the regulation of APA-switching events. Previous studies have demonstrated that the usage of APA could be influenced by the expression level of genes encoding 3′-end-processing factors and the transcription rate of polymerase II [45]. In other studies, differential selection of APA could be ascribed to the strength of different poly(A) signals (weak or strong), and the expression levels of cleavage and polyadenylation specificity factor and cleavage-stimulating factor [49–52]. Furthermore, poly(A)-binding protein nuclear 1, a general factor of polyadenylation, could prevent the usage of proximal poly(A) sites by direct binding to non-canonical poly(A) signals [53–55]. Moreover, the different usage of APA could change the 3’UTR length and content, leading to the loss or gain of regulatory motifs, including miRNA binding sites [20]. In our study, we found that the APA-site switching events could not change the mRNA expression levels, and it could result in the gain or loss of miRNA binding sites in the 3’UTR. For the 119 genes switched to longer 3’UTRs in NPC, totally 706 miRNA targeting sites were gained; and for the 76 genes switched to shorter 3’UTRs, totally 325 miRNA targeting sites were lost. Among them, FNDC3B was identified as the direct and functional target of miR-143 in liver and prostate cancer [56, 57]. In our study, we found that FNDC3B tended to use proximal APA sites and produced mRNAs with shorter 3’UTRs in NPC. Interesting, we noticed that only the longer 3’UTRs harbored the binding sites of miR-143, suggesting that shorter mRNA transcripts can escape from miR-143 regulation. In addition, Zhang et al. reported that *SMAD3* was a direct and functional target of miR-23b [58]. Here, we found that *SMAD3* was prone to using distal APA sites and produced mRNAs with longer 3’UTRs in NPC and using bioinformatics analysis we confirmed that only the longer 3’UTRs harbored the binding sites of miR-23b [59]. These findings provide new insights into how APA mediate the miRNA regulation of gene expression and further affect cell biological function.

## Conclusions

In summary, APA site-switching of 3’UTRs are prevalent in NPC, and APA-mediated regulation of gene expression may play important roles in NPC development and progression. Several GO terms and pathways were enriched in genes that undergo APA-switching events, including regulation of cell migration, macromolecule catabolic process, protein catabolic process, proteolysis, small conjugating protein ligase activity, and ubiquitin-protein ligase activity. These findings suggest that more detailed studies targeted genes undergoing APA site-switching events may provide novel insights into clarifying the pathogenesis of NPC and contribute to the development of novel therapeutic strategies for NPC.

## Additional files

Additional file 1: PCR primers used in quantitative RT-PCR for APA switching genes (PDF 134 kb)

Additional file 2: The frequency distribution of the genes with lengthened 3’UTR (A) and shortened 3’UTR (B) in different pairs, comparing NPC and NNET. (PDF 123 kb)

Additional file 3: Details of the 195 genes with significant differences in 3’UTR lengths between NPC and NNET. (XLSX 96 kb)

Additional file 4: Enrichment of genes with shortened 3’UTR isoforms involved in various GO functional categories. (PDF 144 kb)

Additional file 5: Enrichment of genes with lengthened 3’UTR isoforms involved in various GO functional categories. (PDF 63 kb)

Additional file 6: Details of the 275 genes with significant differences in the mRNA levels between NPC and NNET. (XLSX 94 kb)

Additional file 7: The effect of APA switching events on gene expression. “Blue” and “red” colors indicate up-regulated or down-regulated genes; “Green” and “yellow” colors indicate genes that switched to longer or shorter 3’UTRs. (PDF 200 kb)

Additional file 8: The gain or loss of miRNA binding sites in the 3’UTR of genes with APA-switching events. (XLSX 26 kb)
