# Supplementary material for: Differential genome-wide profiling of alternative polyadenylation sites in nasopharyngeal carcinoma by high-throughput sequencing
Source: J Biomed Sci. 2018 Oct 23;25:74. doi: 10.1186/s12929-018-0477-6 (PMC6198351; doi:10.1186/s12929-018-0477-6)
Supplement: Supplementary file 1 — PCR primers used in quantitative RT-PCR for APA switching genes (PDF 134 kb) [file 12929_2018_477_MOESM1_ESM.pdf]

Additional file 1: PCR primers used in quantitative RT-PCR for APA switching genes

Primers	Sequences
uc002wnw.2 (JAG1)-long-F	5’-GAGGGATGGAGGAAGAAC-3’
uc002wnw.2 (JAG1)-long-R	5’-AATAACAAGGGAATCAAGG-3’
uc002wnw.2 (JAG1)-short-F	5’-TCTCATTACTTGTTGCCTAT-3’
uc002wnw.2 (JAG1)-short-R	5’-CTGCCTCAGTCAGTCCTT-3’
uc003kxa.2 (IRF1)-long-F	5’-TCCAAAGCCAGTGAAATG-3’
uc003kxa.2 (IRF1)-long-R	5’-TAAATGCAAAGGCCAAGA-3’
uc003kxa.2 (IRF1)-short-F	5’-GTTCACATAGACAATGGCAGAA-3’
uc003kxa.2 (IRF1)-short-R	5’-CCAGGAGCCCAGAATCAA-3’
uc001huu.3 (EGLN1)-long-F	5’-TATCTGTGGGTTGTGCTT-3’
uc001huu.3 (EGLN1)-long-R	5’-TAGAGTCCTGCTTGGTGA-3’
uc001huu.3 (EGLN1)-short-F	5’-TAGACAACCAGTTCGCATTT-3’
uc001huu.3 (EGLN1)-short-R	5’-TTCCTCCTGTAAGCAATCAC-3’
uc003anb.2 (TIMP3) –short-F	5’-GCCCTTCTCCTCCAATAC-3’
uc003anb.2 (TIMP3) –short-R	5’-CCTTCCCTCCCTCACTCT-3’
uc003anb.2 (TIMP3) –long-F	5’-AATTGGCTCTTTGGAGGCGA-3’
uc003anb.2 (TIMP3) –longs-R	5’-GGCTAAAGGGAAAGGCGGAT-3’
uc004cey.2 (WDR5)-short-F	5’-GTGTCTTGGGAGTTTGTGG-3’
uc004cey.2 (WDR5)-short-R	5’-TTATTACAGTCTGCGGTGC-3’
uc004cey.2 (WDR5)-long-F	5’-GTCCTTGTGAAGCTCGTCT-3’
uc004cey.2 (WDR5)-long-R	5’-CACCTCCTGTCCAAAGAAAC-3’
uc002aqj.2 (SMAD3)-short-F	5’-GAAGGGCAAGAAATGGCG-3’
uc002aqj.2 (SMAD3)-short-R	5’-AGGGGTGTTTCTGGTTGGAC-3’
uc002aqj.2 (SMAD3)-long-F	5’-GGGTCCTCTGAACCAAGC-3’
uc002aqj.2 (SMAD3)-long-R	5’-GAGTCCAGAACAGCCGAG-3’
uc002vfy.2 (XRCC5)-short-F	5’-GCCATCGCTGTGATGCTG-3’
uc002vfy.2 (XRCC5)-short-R	5’-GGGGTCTAAATTATCCCTTGT-3’
uc002vfy.2 (XRCC5)-long-F	5’-TTCTTGCCTTGAGTTCCA-3’
uc002vfy.2 (XRCC5)-long-R	5’-GACGACTTATGAGGGTGC-3’
uc003fhy.2 (FNDC3B)-short-F	5’-GACTGCCCTAAACAACCAT-3’
uc003fhy.2 (FNDC3B)-short-R	5’-TTGCACAATGCCCTCTTA-3’
uc003fhy.2 (FNDC3B)-long-F	5’-ACTTCAGAACTGCTGCTA-3’
uc003fhy.2 (FNDC3B)-long-R	5’-CCTGGTGGTTAAATGATAG-3’
