# Supplementary material for: Differential genome-wide profiling of alternative polyadenylation sites in nasopharyngeal carcinoma by high-throughput sequencing
Source: J Biomed Sci. 2018 Oct 23;25:74. doi: 10.1186/s12929-018-0477-6 (PMC6198351; doi:10.1186/s12929-018-0477-6)
Supplement: Supplementary file 2 — The frequency distribution of the genes with lengthened 3’UTR (A) and shortened 3’UTR (B) in different pairs, comparing NPC and NNET. (PDF 123 kb) [file 12929_2018_477_MOESM2_ESM.pdf]

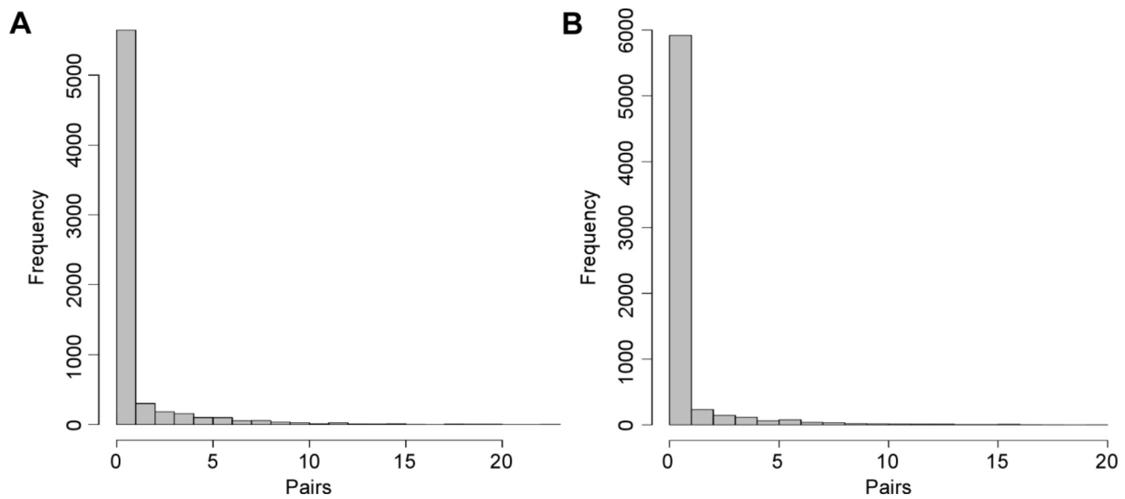


**Additional file 2:** The frequency distribution of the genes with lengthened 3'UTR (A) and shortened 3'UTR (B) in different pairs, comparing NPC and NNET.
