# Supplementary material for: Differential genome-wide profiling of alternative polyadenylation sites in nasopharyngeal carcinoma by high-throughput sequencing
Source: J Biomed Sci. 2018 Oct 23;25:74. doi: 10.1186/s12929-018-0477-6 (PMC6198351; doi:10.1186/s12929-018-0477-6)
Supplement: Supplementary file 4 — Enrichment of genes with shortened 3’UTR isoforms involved in various GO functional categories. (PDF 144 kb) [file 12929_2018_477_MOESM4_ESM.pdf]

Additional file 4: Enrichment of genes with shortened 3'UTR isoforms involved in various GO functional categories.

GO category	Term	Count	P-value
GOTERM_MF_FAT	enzyme binding	8	0.003
GOTERM_MF_FAT	nucleotide binding	17	0.005
GOTERM_BP_FAT	positive regulation of cell migration	4	0.006
GOTERM_BP_FAT	positive regulation of locomotion	4	0.008
GOTERM_BP_FAT	positive regulation of cell motion	4	0.008
GOTERM_BP_FAT	protein catabolic process	8	0.014
GOTERM_BP_FAT	macromolecule catabolic process	9	0.014
GOTERM_MF_FAT	ubiquitin-protein ligase activity	4	0.017
GOTERM_BP_FAT	protein kinase cascade	6	0.018
GOTERM_MF_FAT	purine nucleotide binding	14	0.019
GOTERM_BP_FAT	peptide metabolic process	3	0.020
GOTERM_MF_FAT	small conjugating protein ligase activity	4	0.023
GOTERM_BP_FAT	proteolysis	10	0.028
GOTERM_MF_FAT	kinase binding	4	0.028
GOTERM_BP_FAT	cellular macromolecule catabolic process	8	0.029
GOTERM_MF_FAT	purine ribonucleotide binding	13	0.032
GOTERM_MF_FAT	ribonucleotide binding	13	0.032
GOTERM_BP_FAT	regulation of cell migration	4	0.033
GOTERM_BP_FAT	protein oligomerization	4	0.035
GOTERM_MF_FAT	protein complex binding	4	0.036
GOTERM_BP_FAT	proteolysis involved in cellular protein catabolic process	7	0.037
GOTERM_BP_FAT	cellular protein catabolic process	7	0.038
GOTERM_MF_FAT	acid-amino acid ligase activity	4	0.038
GOTERM_MF_FAT	insulin receptor substrate binding	2	0.040
GOTERM_BP_FAT	regulation of locomotion	4	0.045
GOTERM_BP_FAT	regulation of cell motion	4	0.046
KEGG_PATHWAY	Lysosome	4	0.021
KEGG_PATHWAY	Ubiquitin mediated proteolysis	4	0.032
