# Supplementary material for: Differential genome-wide profiling of alternative polyadenylation sites in nasopharyngeal carcinoma by high-throughput sequencing
Source: J Biomed Sci. 2018 Oct 23;25:74. doi: 10.1186/s12929-018-0477-6 (PMC6198351; doi:10.1186/s12929-018-0477-6)
Supplement: Supplementary file 5 — Enrichment of genes with lengthened 3’UTR isoforms involved in various GO functional categories. (PDF 63 kb) [file 12929_2018_477_MOESM5_ESM.pdf]

Additional file 5: Enrichment of genes with lengthened 3'UTR isoforms involved in various GO functional categories.

GO category	Term	Count	P-value
GOTERM_BP_FAT	RNA processing	10	0.008
GOTERM_BP_FAT	RNA splicing	7	0.009
GOTERM_BP_FAT	RNA splicing, via transesterification reactions	5	0.016
GOTERM_BP_FAT	nuclear mRNA splicing, via spliceosome	5	0.016
GOTERM_BP_FAT	regulation of binding	5	0.016
GOTERM_BP_FAT	RNA splicing, via transesterification reactions with bulged adenosine as nucleophile	5	0.016
GOTERM_BP_FAT	mRNA processing	7	0.016
GOTERM_BP_FAT	regulation of cell migration	5	0.023
GOTERM_BP_FAT	skin morphogenesis	2	0.025
GOTERM_BP_FAT	regulation of cytokine production	5	0.028
GOTERM_BP_FAT	mRNA metabolic process	7	0.030
GOTERM_BP_FAT	regulation of locomotion	5	0.034
GOTERM_BP_FAT	regulation of cell motion	5	0.035
GOTERM_MF_FAT	transcription repressor activity	6	0.036
KEGG_PATHWAY	Spliceosome	4	0.047
