# Supplementary material for: Differential genome-wide profiling of alternative polyadenylation sites in nasopharyngeal carcinoma by high-throughput sequencing
Source: J Biomed Sci. 2018 Oct 23;25:74. doi: 10.1186/s12929-018-0477-6 (PMC6198351; doi:10.1186/s12929-018-0477-6)
Supplement: Supplementary file 7 — The effect of APA switching events on gene expression. “Blue” and “red” colors indicate up-regulated or down-regulated genes; “Green” and “yellow” colors indicate genes that switched to longer or shorter 3’UTRs. (PDF 200 kb) [file 12929_2018_477_MOESM7_ESM.pdf]

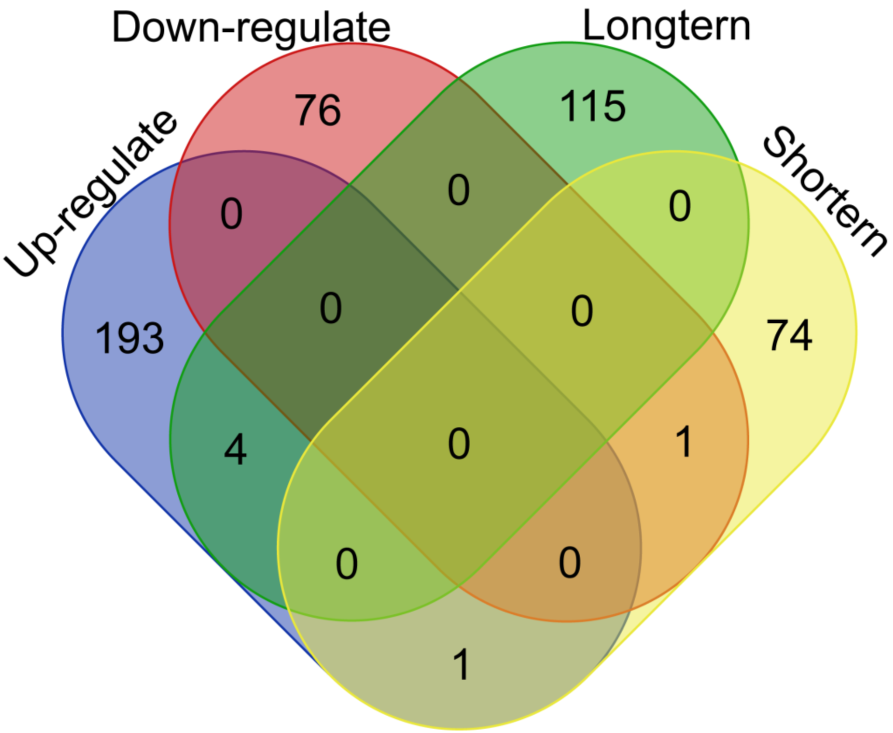

Additional file 7: The effect of APA switching events on gene expression. "Blue" and "red" colors indicate up-regulated or down-regulated genes; "Green" and "yellow" colors indicate genes that switched to longer or shorter 3'UTRs.
